# TSMG: A Deep Learning Framework for Recognizing Human Learning Style Using EEG Signals

**DOI:** 10.3390/brainsci11111397

**Published:** 2021-10-24

**Authors:** Bingxue Zhang, Yang Shi, Longfeng Hou, Zhong Yin, Chengliang Chai

**Affiliations:** 1 Department of Optical-Electrical & Computer Engineering, University of Shanghai for Science and Technology, Shanghai 200093, China; zhangbingxue@usst.edu.cn (B.Z.); 193780684@st.usst.edu.cn (Y.S.); yinzhong@usst.edu.cn (Z.Y.); 2 Department of Energy & Power Engineering, University of Shanghai for Science and Technology, Shanghai 200093, China; longfenghou@126.com

**Keywords:** learning style, EEG signal, deep learning, one-dimensional spatio-temporal convolution, multi-scale feature extraction

## Abstract

Educational theory claims that integrating learning style into learning-related activities can improve academic performance. Traditional methods to recognize learning styles are mostly based on questionnaires and online behavior analyses. These methods are highly subjective and inaccurate in terms of recognition. Electroencephalography (EEG) signals have significant potential for use in the measurement of learning style. This study uses EEG signals to design a deep-learning-based model of recognition to recognize people’s learning styles with EEG features by using a non-overlapping sliding window, one-dimensional spatio-temporal convolutions, multi-scale feature extraction, global average pooling, and the group voting mechanism; this model is named the TSMG model (Temporal-Spatial-Multiscale-Global model). It solves the problem of processing EEG data of variable length, and improves the accuracy of recognition of the learning style by nearly 5% compared with prevalent methods, while reducing the cost of calculation by 41.93%. The proposed TSMG model can also recognize variable-length data in other fields. The authors also formulated a dataset of EEG signals (called the LSEEG dataset) containing features of the learning style processing dimension that can be used to test and compare models of recognition. This dataset is also conducive to the application and further development of EEG technology to recognize people’s learning styles.

## 1. Introduction

### 1.1. Field Overview

#### 1.1.1. Learning Style

A person’s learning style is a relatively stable preference [1] in long-term learning activities, and is an important factor reflecting differences between learners. By analyzing the differences in people’s learning styles, and providing personalized learning strategies and resources to them, their efficiency of learning as well as capacity for independent learning can be improved [2]. If the teacher can understand the student’s learning style and provide a learning plan suitable for them, their academic performance can significantly improve (e.g., the teacher can provide the time needed by reflective learners for independent learning, and can supply the relevant discussion-based learning plans for active learners). This idea is widely applied in educational circles in various countries [3]. In a survey of teachers from the UK, Netherlands, Turkey, Greece, and China, researchers found that 95% of teachers agreed that when students receive teaching strategies that are consistent with their learning styles, they usually learn better [3].

Classic models of styles of learning include the Felder–Silverman model [4], Kolb model [5], and VARK [6]. Of these, the Felder–Silverman model is the most widely applied [7]. It conceptualizes two groups of learning styles according to the learners’ preferences in four dimensions: information perception, input, understanding, and processing [7].

#### 1.1.2. Existing Methods to Recognize Learning Style

Traditional methods to recognize learning styles are mostly based on the index of learning styles (ILS) questionnaires [8,9] and online behavior analyses [10,11]. On one hand, learners will have their own subjectivity when filling out the ILS. Questions on the ILS are relatively abstract and thus difficult to understand. On the other hand, it is also difficult to obtain a complete user portrait from a single online learning and education system, and this hinders the accurate recognition of people’s learning styles through user behavior. Therefore, such non-physiological signals are not effective and reliable in applications [12].

#### 1.1.3. Relationship between Learning Style and EEG

Many studies indicate electrophysiological correlates of the learning process [13,14]. Besides, there are two findings that also provide information on how individual differences in cognitive abilities, such as working memory, are crucially involved in the learning process [15,16]. Differences in the dimensions of information processing, perception, input, and understanding in the context of the style of learning manifest the differences among people in analyzing and solving problems [17]. This process is related to internalization and information comprehension in humans, and is challenging to efficiently analyze through the conventional methods of recognition described above. Neural signals are innervated by the central nervous system, and are not easily controlled by the subjective consciousness, and can thus reflect people’s objective internal state [18,19]. Research in neuroscience and psychology has shown that neural signals are more objective reflections of mental activity and cognition than behaviors, facial expressions, voices, and so on [20,21]. Therefore, they are difficult to disguise, are in real time, and are more accurate than other methods based on signal recognition. Electroencephalography (EEG) [22], magnetic resonance imaging (MRI) [23], and functional MRI (fMRI) [24] have been widely used to non-intrusively detect brain activity. When comparing these three methods [25], it is clear that EEG has advantages in terms of its temporal resolution of brain activity, the directness of measuring brain activity, and portability. These advantages make it more conducive than the other two techniques to recognizing people’s internal learning style. Therefore, it offers promise for decoding the brain. This study uses features of EEG signals to recognize people’s learning styles.

#### 1.1.4. Basic Process of EEG Data Processing

**Figure 1:**
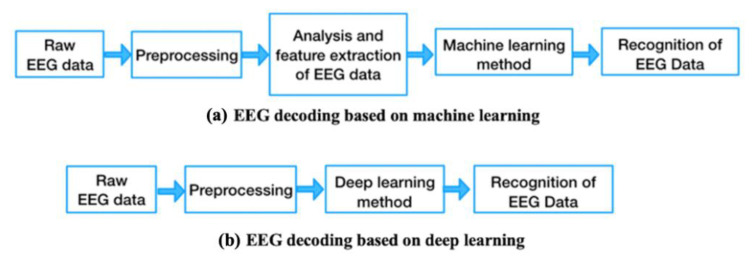
Experimental flowchart of EEG signal processing based on machine learning and deep learning.

An experimental flowchart of EEG signal processing based on machine learning and deep learning is shown in Figure 1. As shown in Figure 1a, EEG signal recognition based on traditional machine-learning-based methods includes the preprocessing of the EEG signals, feature extraction, and training and classification of the target patterns (for instance, by using the support vector machine (SVM), k-nearest neighbor (KNN), or Bayesian network (BN)). The process relies on manual feature extraction for classification, which makes it difficult to improve its accuracy of classifying EEG signals. As shown in Figure 1b, a deep neural network can automatically learn EEG signals in an end-to-end manner, extract their features, and classify them. The deep networks used for EEG recognition include the convolutional neural network (CNN), recurrent neural network (RNN), and hybrid network architectures. EEG signal recognition based on deep learning can not only learn manually extracted features, but also can automatically learn features from the original EEG signals or two-dimensional (2D) images that have been transformed from the frequency domain. Therefore, deep learning technology offers greater promise than traditional machine-learning-based methods of processing for the recognition and classification of EEG signals [26].

#### 1.1.5. Experiment to Recognize Learning Style Using EEG Features

**Figure 2:**
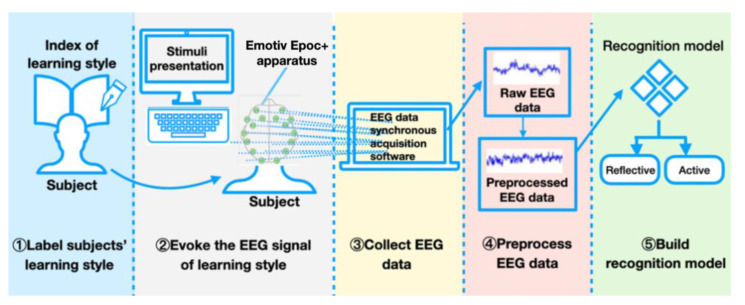
Procedure of experiment to recognize learning styles.

An experiment to recognize learning styles is shown in Figure 2. The detailed experimental process has been provided in our past work [27]. The previous research verified the design and feasibility of the learning style experimental process method without further experiments or in-depth discussion on the structure and accuracy of the recognition model. Later, we found that the whole experiment still needs to be discussed and verified at the algorithm level. Therefore, based on the experimental part of our previous article [27], we proposed a new deep learning model for the field of learning style recognition with EEG features, and conducted a large number of experimental designs to verify the superiority of the proposed recognition model in the field of learning style recognition with EEG features from multiple dimensions. At the same time, these experimental verification methods can also provide reference for the evaluation of other recognition models in other fields with EEG features.

(1) Labeling subjects’ learning style: We asked the subjects about their willingness to fill out the ILS in advance, and only those who expressed willingness to do so were selected. We translated each ILS item in a straightforward, detailed manner, and explained the meaning of each item to the subjects before they filled it out. They were asked to fill out the ILS based on careful consideration of their own actual situation. On this basis, subjects’ learning styles were obtained, providing a reliable basis for labeling learning styles.
(2) Evoking the EEG signal of the learning style: For the selection of stimuli, Raven’s Advanced Progressive Matrices (RAPM) is selected. RAPM asks subjects to think logically based on the rules associated with the symbols in the matrix diagram; RAPM test questions are shown in [28]. RAPM can not only effectively stimulate the differences in the subjects’ learning styles in the processing dimension, but can also ensure that the designed stimulus mode would generate as few invalid signals as possible. Using RAPM as a stimulus can prompt subjects to undertake logical thinking, which will stimulate brain processing.
(3) Collecting the EEG data: The subjects wear a brain–computer device so that their EEG data can be recorded by one computer. The Emotiv Epoc+ is used because it is lightweight and easy to use, which can reduce the stress or nervousness of subjects in the study and provide a better setup while still delivering reliable results [29]. A computer is used to present a stimulus to the subject, and another computer is used to simultaneously record their EEG signals.
(4) Processing EEG data and building recognition model: The collected EEG raw data are preprocessed by the EEG processing methods (including removing the unused frequency range, EOG and EMG artifacts, etc.), and then the preprocessed EEG data will be inputted into the recognition model (e.g., machine learning methods, deep learning methods) to recognize the subjects’ learning styles.

### 1.2. Literature Review

#### 1.2.1. Recognition of Learning Style

There are two main ways of recognizing learning style. The first is explicit recognition, that is, using the Index of Learning Styles (ILS) [30]. Scores on the ILS are used to identify the subjects’ learning styles [31]. The models to identify learning style developed by Surjono [8], Yang [9], and Wang [32] are all based on subjects filling out the ILS, which is customized according to the authoritative model of learning. This method has been shown to be reliable, and has theoretical support, but learners struggle to understand the learning styles and concepts of the ILS, and thus may not be able to accurately answer its questions. Moreover, they exhibit a subjective bias toward the results when filling out the ILS, and this can affect the objectivity of the outcomes. Further, the learning style identified through a one-time calculation of the ILS cannot reflect its changing characteristics over time.

The second method used to recognize the learning style is implicit recognition, that is, by mining and analyzing data concerning the learners’ interactive behavior on online learning systems, including through logs of learning behavior and data on social behavior. This is used to indirectly identify learning styles without the use of the ILS. A number of researchers have studied implicit recognition. Cha et al. [10] used online data regarding interactive behavior (such as the number of clicks on specific buttons, time taken for learning activity, test scores, and the number of posts written and read in the forum as input sources) on a decision tree and hidden Markov model to train and recognize people’s learning styles, respectively. Villaverde et al. [11] used the following data as input: the learners’ preferred types of learning materials, whether they actively engaged with the given learning module, and whether they modified their answers before submitting them. They used an artificial neural network (ANN) for training and recognition. This solves the problem of subjectivity encountered in explicit recognition, and saves learners the time needed to fill out the ILS questionnaire. However, implicit recognition encounters the problem of a "cold start," which means that a large amount of data on online learning behavior is needed to accurately identify a learner’s learning style.

#### 1.2.2. Application of EEG

The characteristics of EEG signals have been widely used for emotion recognition [33,34]; measuring attention levels [35] and cognitive load [36,37]; detecting states of cognition [38,39], academic stress [40], cognitive mental diseases [41,42], motor imagery [43,44], and music preference [45]; fatigue monitoring [46] and mind control [47].

#### 1.2.3. Processing Variable-Length EEG Data

Because the length of EEG records collected in acquisition experiments is usually variable, the sliding window is used to process these data [48,49]. In [48], Liu et al. proposed a fractal dimensions-based basic method of emotion quantification based on a sliding window. In [49], Xu et al. proposed the MW-TFA method, which uses a set of sliding windows to process EEG data instead of a single sliding window. Experimental results showed that the MW-TFA technology can predict the time–frequency distribution of the data. However, the traditional sliding window generates repeated calculations in overlapping parts of the data to incur a large computational cost (large number of FLOPs) and long operation time that degrade the efficiency of the model. This limits its use in large-scale-use scenarios.

#### 1.2.4. Methods to Recognize EEG Data

The algorithms used to recognize EEG data can be divided into traditional machine-learning-based and end-to-end algorithms.

In traditional classifiers of EEG data, the data are filtered in the temporal, frequency-based, or spatial domain to extract their features. However, traditional methods rely on domain-specific knowledge and use manual feature extraction to establish a robust classifier [26]. Li et al. [50] applied 18 kinds of linear and nonlinear features to study emotion recognition, and achieved accuracies of 59.06% and 83.33% on two public datasets. Of traditional machine learning classifiers, the SVM has been widely used for EEG signal processing [48,51,52]. Atkinson et al. [52] proposed a feature selection method that improves the performance of the SVM classifier in terms of detecting emotional arousal. Tsoi et al. [53] used an artificial neural network (ANN) to recognize patients with mental diseases using EEG data. Although many machine learning methods have been proposed for EEG data recognition, most of them are highly dependent on manual feature extraction, which is time consuming, and this affects the performance of the classifier.

In recent years, deep learning networks have achieved promising results in classifying EEG signals [54]. Such classifiers use a deep neural network for feature extraction, and yield good classification performance. Li et al. [55] proposed a hierarchical convolutional neural network (HCNN) to extract spatial information on EEG electrodes by mapping EEG signals to a 2D position map. Zheng et al. [21] used the discriminant graph-regularized extreme learning machine with DE features to study stable patterns of EEG data over time for emotion recognition. Lawhern et al. [56] proposed an end-to-end deep learning framework called EEGNet that can extract hidden spatio-temporal patterns from raw EEG data. León et al. proposed an accuracy/cost trade-off strategy of deep learning method for EEG-based motor imagery classification [57]. Yang et al. [58] designed a hierarchical network structure with sub-network nodes to classify EEG data with emotional characteristics. However, current end-to-end models cannot consider both local and global features at the same time, and their feature extraction strategies are inadequate. This leaves considerable room for improvements to them.

### 1.3. Problem Focus and Solution

(1) How do we deal with variable-length data more efficiently?

The time needed to stimulate each learner’s internal state is different when recognizing different learning styles. When facing the same test question, each learner takes a different duration to think about it, and this is reflected in the different times taken to answer the question by different people. Therefore, the length of EEG records collected in experiments to recognize learning styles is usually variable, and dealing with such input data is an important problem. The input data used by traditional classifiers is fixed in length, therefore they need to limit all EEG records to a fixed duration. However, this leads to a loss of information on hidden features of the data. Current methods used to process variable-length EEG data encounter the problem of a large cost of calculation that influences performance. Efficiently processing variable-length data can improve the efficiency of calculation of the model and loosen the limitations on EEG data collection. We use a non-overlapping sliding window here that can process information of any length, extract the characteristics of each data segment, and reduce computational complexity to improve the efficiency of calculation.

(2) How do we reduce the cost of calculation while increasing accuracy?

Reducing the amount of requisite calculation and improving the accuracy of the model are key to optimizing its performance. Designing a model with a reasonable structure can improve its performance as well. Inspired by the field of computer vision, this paper proposes an optimized CNN model to identify learning styles called the TSMG model. The spatial and temporal convolution operations are first used in it to extract the temporal and spatial characteristics of EEG signals. At the same time, a 1D convolution is used to reduce the number of parameters and cost of calculation of the model. The capability of the model for feature abstraction is then enhanced by constructing a multi-scale convolution, and the global average pooling strategy is used to reduce the number of training parameters. The limitation on the size of the input data, which plagues traditional methods, is overcome to enable the processing of variable-length data. Finally, the accuracy of recognition of the model is improved by using the group voting mechanism, while ensuring a low cost of calculation. The TSMG model also provides a new idea for optimizing models used to recognize EEG data.

(3) The absence of an EEG dataset on learning styles

A dataset of EEG data related to learning styles can not only help researchers measure the pros and cons of models of recognition, but also can help develop new models to identify learning styles. We develop an EEG dataset here, called the LSEEG dataset, that contains characteristics of people’s learning styles that were collected through experiments. The LSEEG dataset can be used to study the characteristics of EEG signals for different learning styles.

### 1.4. Highlights

(1) We design a deep learning model (TSMG) by using a non-overlapping sliding window, 1D spatio-temporal convolutions, multi-scale feature extraction, global average pooling, and the group voting mechanism for recognizing the features of EEG signals to solve the problem of processing variable-length EEG data. The proposed model improves the accuracy of recognition by nearly 5% compared to prevalent methods, while reducing the amount of calculations needed by 41.93%. The model can also recognize variable-length data in other fields.
(2) We develop an EEG dataset (LSEEG dataset) containing features of the learning styles in processing dimensions. It can be used for testing and comparing models for the recognition of learning styles, and can help with the application and further development of EEG technology in the context of identifying learning styles.

## 2. Methodology

### 2.1. Review of Basic Knowledge

The convolutional neural network (CNN) can automatically learn and extract the features of EEG data, avoiding the complicated process of manual feature extraction, and thus is a popular subject of research in EEG decoding [46]. The CNN consists of a convolutional layer, a down-sampling layer, and a fully connected layer. The training process uses the convolutional layer to extract features from the data, and the fully connected layer and the classifier are used to output the results of recognition. The convolution is calculated as follows:

(1) $$x_{j}^{l}=\delta \left(\underset{i\in M_{j}}{\sum }x_{i}^{l-1}\omega_{ij}^{l}+b_{j}^{l}\right)$$

where $x_{j}^{l}$ is the *j*-th feature of the *l*-th layer, $\omega_{ij}^{l}$ is the weight of the connection between the *j*-th feature of the *l*-th layer and the *i*-th feature of the *l*-1th layer, $b_{j}^{l}$ is the corresponding bias, and *δ* (·) is the activation function.

The traditional CNN model is poor at recognizing EEG signals because it cannot fully extract their temporal and spatial features. To solve this problem, we construct an optimized CNN model that can recognize the learning style of a subject as represented by their EEG signals in the temporal and spatial domains to improve the accuracy of recognition.

### 2.2. General Structure of Proposed TSMG Model

**Figure 3:**
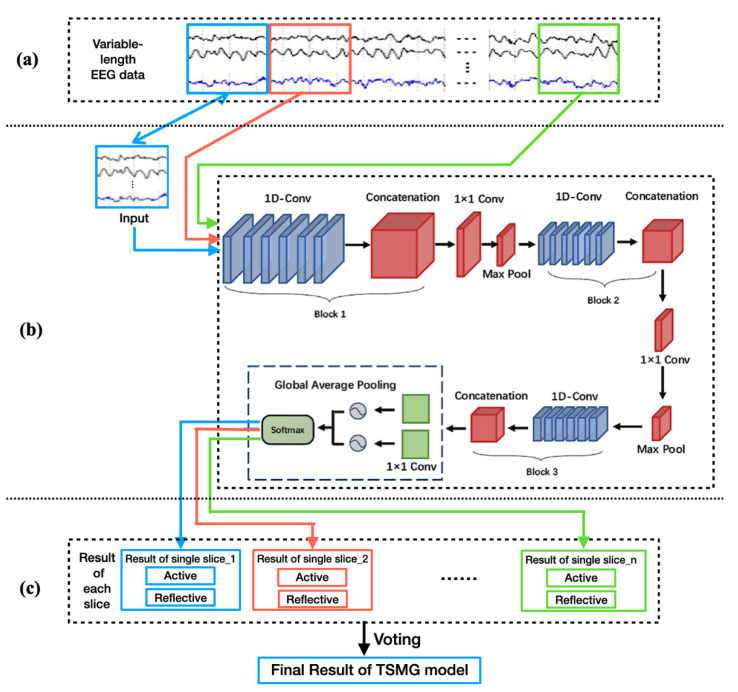
Proposed model to recognize learning styles. (**a**) Non-overlapping sliding window; (**b**) Deep learning modules of TSMG model; (**c**) Group voting mechanism.

A schematic diagram of the TSMG model is shown in Figure 3. It is composed of five modules: a non-overlapping sliding window, a 1D spatio-temporal convolution, multi-scale feature extraction, global average pooling, and the group voting mechanism.

As shown in Figure 3a, we use a non-overlapping sliding window to process variable-length EEG data to ensure that data of any size can be used by our recognition model. Figure 3b shows that the model is composed of three multi-scale feature extraction modules. Features of the input data at multiple scales are extracted to yield six-branch feature maps, which are then subjected to feature fusion. The feature map is processed by a 1 × 1 convolution layer and the max pooling layer, and the results of dimension reduction are inputted into the next feature extraction module. After repeating this procedure on the training samples three times, a generalized feature output is obtained. This is entered into the Softmax layer to obtain the results of classification and recognition of a single slice. As shown in Figure 3c, the result of recognition of a single segment is processed using the group voting module, which outputs the final result.

### 2.3. Non-Overlapping Sliding Window

To solve the problem of variable-length data as input and increase the number of training samples, the non-overlapping sliding window is used to slice the raw data. Its operation is shown in Figure 3a. The variable-length EEG data are first divided into multiple data slices—slice (1) to slice (n)—over 2 s by using the sliding window. There is no overlap between the data slices; those that appear sooner than 2 s are ignored. This method can be used to process EEG data of any length, extract the characteristics of the data slices, and reduce computational complexity to improve the computational efficiency of the model. The data, after being sliced, are used as the input data to the TSMG model to identify people’s learning styles.

### 2.4. 1D Spatio-Temporal Convolutional Layer

**Figure 4:**
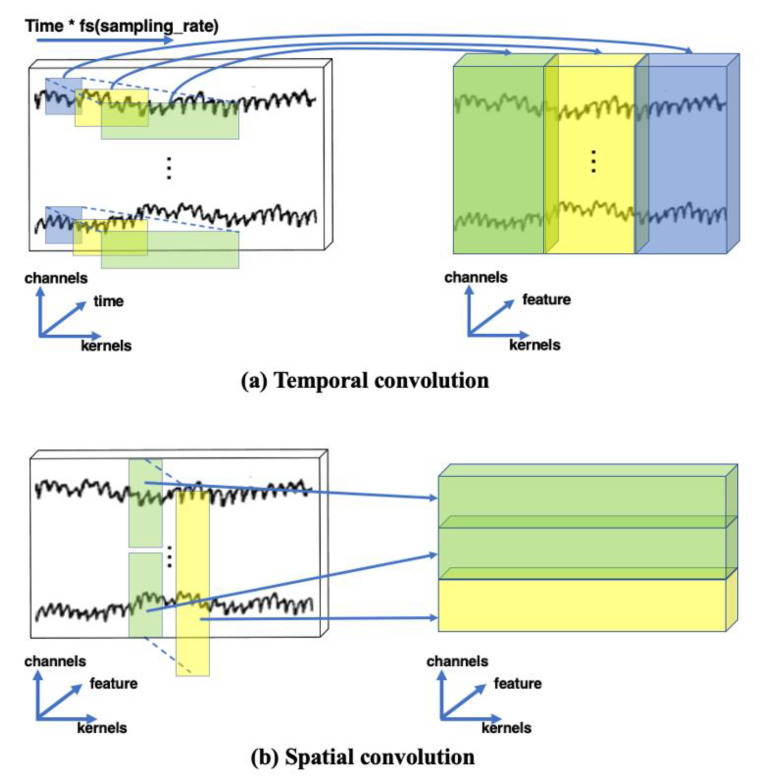
Examples of temporal and spatial convolutions.

To make full use of the temporal and spatial features of the EEG signals, the proposed TSMG uses a 1D convolutional layer to extract them. A 1D convolution is often used to model a temporal series because it can extract features from shorter data fragments and has advantages in terms of processing. According to the different dimensions of feature extraction, the TSMG model simultaneously uses the following two 1D convolutional layers for feature extraction:

(1) Temporal convolution: As shown in Figure 4a, the 1D convolution is calculated on different channels of the original EEG signals along the time axis, and the output is the temporal features of the EEG signals containing different bandpass frequencies, which are suitable for frequency recognition over a short time scale.
(2) Spatial convolution: As shown in Figure 4b, the spatial convolution is a convolutional filter acting on the channel that extracts the characteristics of spatial distributions of different channels. The spatial convolution is also often used to decompose the convolution operations to reduce the number of training parameters and the time needed to train the model.

### 2.5. Multi-Scale Feature Extraction Module

To improve the ability of the convolutional layer to extract EEG signals and obtain richer input features, we design a single-layer multi-scale feature extraction structure. Multiple convolution kernels of different sizes are used in the same layer of the convolution operation, and the multi-scale features are fused as the output and calculated in the next layer. The structure and the parameters of the multi-scale feature extraction module are as shown in Table 1. It can enhance the model’s adaptability to scale, increase the bandwidth of the network, and improve the generalization ability of the network. The input layer accepts the raw EEG signal and passes through four parallel feature extraction branches, where each branch contains several spatial and temporal convolutional layers of different sizes. The model selects three temporal convolutional layers of sizes 1 × 3, 1 × 5, and 1 × 7, as well as the spatial convolutional layers of the corresponding scales. The 1 × 1 convolution is added before the 1 × 7 and 1 × 5 temporal convolution layers to reduce the number of dimensions of the features. The final 1 × 1 convolution branch reduces the dimensionality of the original input so that more features of the original EEG signal can be retained. Each convolution layer is followed by batch normalization (BN) and a ReLU layer. The BN layer is used to speed up training and the ReLU layer to enhance the nonlinear capability of the model. The number of convolution kernels doubles with each increase in the number of layers. Finally, 40 layers of feature maps are obtained for each branch. After the convolution layer, the outputs of all branches are fused, and the fused features are outputted to the next feature extraction module after dimension reduction.

### 2.6. Global Average Pooling

**Figure 5:**
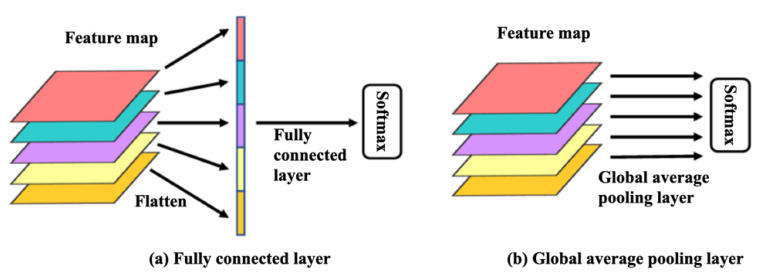
Comparison of the fully connected layer and global average pooling layer.

The third module of the TSMG is global average pooling (GAP). The traditional convolutional neural network processes the output of vectorization through the fully connected layer (FC), as shown in Figure 5a. However, the FC layer usually contains a large number of training parameters, which slows the speed of model training; in addition, it can receive only input parameters of a fixed length, and it can process data at any scale. The proposed TSMG model uses a GAP layer instead of the FC layer. As shown in Figure 5b, the GAP layer maps each feature map to a single feature value through an average pooling operation, and the feature value can be directly inputted to the Softmax classifier. Therefore, the GAP reduces the number of training parameters of the model, minimizes the overfitting effect, and overcomes the requirement of the FC layer for a fixed-parameter input so that any scale of data can be processed.

### 2.7. Group Voting Mechanism

The last part of the TSMG algorithm is the group voting mechanism, which is designed based on the idea that “the collective decision-making ability is greater than individual decision-making ability” [59]. Once the results of prediction of a single classifier are placed in the ballot box, the label of the winning category is determined according to the number of votes.

In the TSMG model, the group voting mechanism is carried out according to the results of each question, and the result of voting is taken as the final result of the learning style that improves the accuracy of recognition of the TSMG. In addition, to ensure that the final result is odd, when the result of the given slice is even, the last piece of data is discarded so that the result of voting is not in both the "active" and the "reflective" states at the same time, and only one state is generated.

## 3. Proposed EEG Dataset—LSEEG Dataset

The procedure of the experiment used to identify learning styles has been described in Section 1.2. All experimental details have been described in our past study [27]. In this paper, we used EEG data to verify our TSMG method.

### 3.1. Details of LSEEG Dataset

The Emotiv Epoc+ [60], a wireless EEG instrument with non-invasive electrodes that was developed by Emotiv Systems, was used for data collection to form the LSEEG dataset. The device had 14 data acquisition channels, its sampling rate was 128 Hz, and the electrodes were arranged in accordance with the international 10–20 standard electrode placement method. The environment for data collection was a quiet and comfortable laboratory. A total of 14 subjects participated in this experiment (age range: 18–21 years old, average age: 19.4 years old), including seven reflective learners and seven active learners (each subject showed a prominent active or reflective learning style). Each subject completed 36 test questions to stimulate the learning style in the processing dimension. A total of 504 items of EEG data on learning styles (duration: 5 s to 60 s) were thus collected. We publish the LSEEG dataset in this paper, and readers can download the data freely from https://github.com/aegine-lab/dataset, accessed on 23 October 2021. To our best knowledge, this is the first release that can be used to build a practical learning style recognition system. We hope this release can provide a standard reference for all the researchers who are interested in learning styles.

### 3.2. Visualization of EEG Responses of LSEEG Dataset

**Figure 6:**
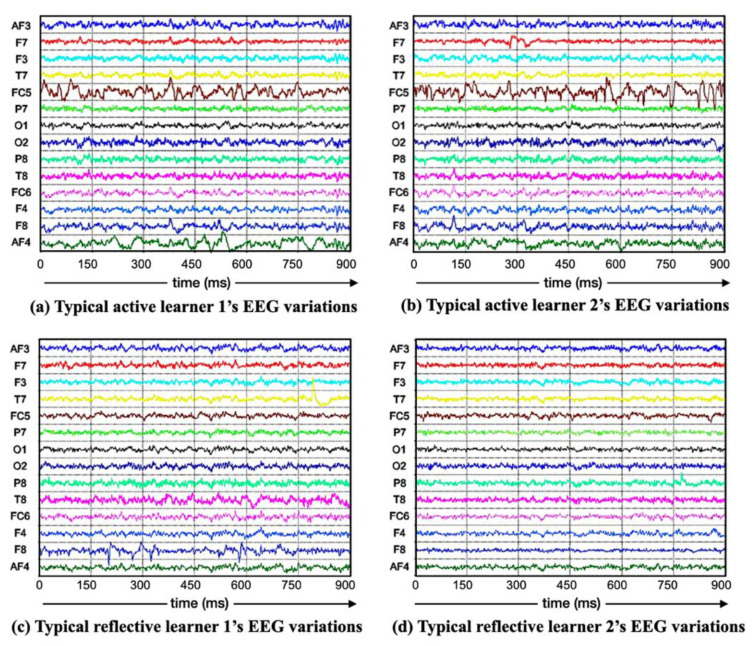
Visualization of EEG responses of LSEEG dataset.

To present the real EEG data intuitively, the variations in EEG responses of four typical learners on the same test questions are plotted for the visualization corresponding to all 14 electrodes, which can be seen in Figure 6. The abscissa represents the time span of the EEG waveforms. The ordinate represents the EEG waveform collected by each electrode. The amplitude range of each electrode in Figure 6 is “−50µV~50µV”. Figure 6a,b are two active learners’ EEG variations, and Figure 6c,d are two typical reflective learners’ EEG variations.

### 3.3. Two-Tailed Paired t-Test on EEG Responses of LSEEG Dataset

In order to reflect the internal difference of EEG responses between the two types of learners with different learning styles from the LSEEG dataset. A two-tailed *t*-test analysis [61] of the difference between the two types of subjects in reflection time and the test scores during the process of EEG data collection is performed.

A significant difference was found on the actual performance of reflection time between active learners and reflective learners during the data collection period, with *t* = 3.2470 and *p* = 0.0070, and effect size (based on Cohen’s d = 1.8744) of 0.6838. In addition, there was a great significant difference found on the test scores between active learners and reflective learners, with *t* = 2.3127, *p* = 0.0393, and effect size (based on Cohen’s d = 1.3352) of 0.5552. Therefore, the *p*-value of the two types of subjects in reflection time and the test scores are both less than 0.05. According to the statistical significance analysis of the two tailed *t*-test in [61], this indicates that the EEG response contained in the LSEEG data set contains significantly different characteristics in the dimension of learning style.

## 4. Parameter Setting and Model Training

The procedure of the experiment used to identify learning styles has been described in Section 1.1.5. All experimental details have been described in our past study [27]. In this paper, we used EEG data to verify our TSMG method.

### 4.1. Parameter Setting of TSMG Model

The parameters of the TSMG model can be described as follows:

(1) Learning rate: The learning rate affects the convergence of the model [62]. In this paper, the decay method for the learning rate was chosen. The idea is to let the learning rate gradually decay with training. The algorithm is as follows:

(2) $$\alpha =\frac{1}{1+\sigma n}\alpha_{0}$$

where σ is the decay rate, n is the training algebra, and $\alpha_{0}$ is the initial learning rate. In this paper, $\alpha_{0}$ was initialized to 0.1, and σ was set to 0.2.

(2) Loss function: The loss function is used to measure the performance of the model [62]. Cross-entropy loss was used in this paper. It the most commonly used loss function for classification tasks, and is defined as follows:

(3) $$J=-\frac{1}{N}\overset{N}{\underset{1}{\sum }}\overset{k}{\underset{i=1}{\sum }}y_{i}log\left(p_{i}\right)$$

where $y_{i}$ is the true label of category *i*, $p_{i}$ is the probability of category *i* being calculated by the Softmax classifier, *k* is the number of categories, and *N* is the total number of samples.

(3) Optimizer: The optimizer minimizes loss so that parameter update is not affected by the change in the scale of the gradient. Its formula is as follows:

(4) $$m_{t}=\beta_{1}m_{t-1}+\left(1-\beta_{1}\right)g_{t}$$

(5) $$v_{t}=\beta_{2}v_{t-1}+\left(1-\beta_{2}\right)g_{t}{}^{2}$$

where $g_{t}$ is the gradient at time *t*, $\beta_{1}$ and $\beta_{2}$ are exponential decay rates, $m_{t}$ represents the estimated value of the first momentum of the gradient, and $v_{t}$ represents the estimated value of the second momentum of the gradient. The formula for update is as follows:

(6) $$\theta_{t+1}=\theta_{t}-\frac{\eta }{\sqrt{\frac{v_{t}}{1-\beta_{2}{}^{t}}}+\epsilon }\frac{m_{t}}{1-\beta_{1}{}^{t}}$$

where *η* is the learning rate, *ϵ* is a constant, and $\theta_{t}$ is the initial parameter. In this paper, $\beta_{1}$ was initialized to 0.9, $\beta_{2}$ to 0.999, and *ϵ* was 10e^−8^.

### 4.2. Training Process of TSMG Model

Before model training, 80% of the 8,358 training data items were sampled as the training set, and the other 20% as the testing set. There was no overlap between the datasets. The process of model recognition consisted of two parts: training and testing.

In the training phase, the model extracted features and updated the parameters of the preprocessed EEG signals. In the testing phase, the test dataset was used to evaluate the performance of the model.

**Figure 7:**
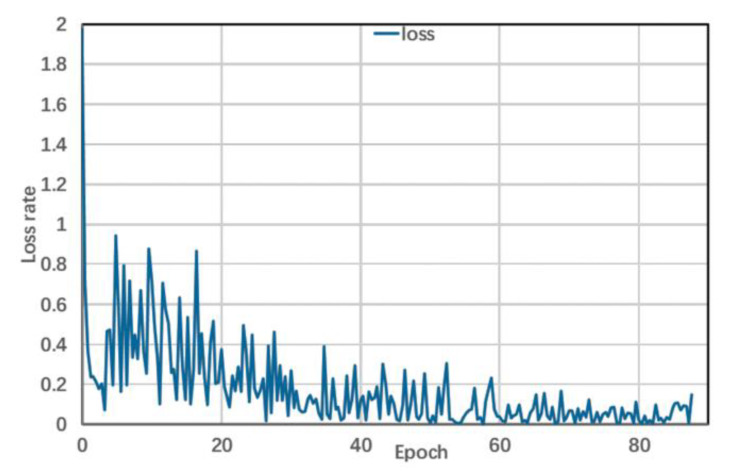
Variation of model loss with the number of epochs.

During training, the number of epochs was set to 100, and early stopping was used to optimize the model. Figure 7 shows variations in losses of the model with the number of iterations. When the number of training epochs was 60, the loss tended to be flat and did not decrease further. At this time, training was stopped, and the weight after stopping was taken as the final parameter of the model.

### 4.3. Parameter Setting and Training of Models for Comparison

#### 4.3.1. Feature Extraction of the Compared Models

We chose 14 EEG acquisition channels: F3, F4, AF3, AF4, F7, F8, P7, P8, FC5, FC6, T7, T8, O1, and O2. For each data segment, 174 EEG features were extracted, consisting of 76 features of the frequency domain and 98 from the time domain. The frequency-related features (56 power features, 20 power difference features) were processed by using the fast Fourier transform.

In each channel, the power-related features were computed on four frequency bands: theta (4–8 Hz), alpha (8–12 Hz), beta (12–30 Hz), and gamma (30–45 Hz). The power-difference-related features were applied to detect the variation in cerebral activity between the left and right cortical areas. Five channel pairs—F4-F3, AF4-AF3, T8-T7, P8-P7, and O2-O1—were used to extract the differences in power, with each pair contributing four features of the four bands. The remaining 98 features consisted of the mean, variance, zero crossing rate, Shannon entropy, spectral entropy, kurtosis, and skewness across the 14 channels. All EEG features were standardized to zero mean and unit variance. A detailed description of the features is provided in Table 2.

#### 4.3.2. Parameter Setting of the Compared Models

The SVM, BP, KNN, VGGNet, and ResNet were used to recognize the same LSEEG dataset, and their results were compared with those of the proposed model.

Details of the experimental settings for classification used in the subsequent sections are listed in Table 3. A number of classification methods were analyzed, including the SVM [63], k-nearest neighbors (KNNs) [64], back-propagation network (BP) [65], VGGNet [66], and ResNet [67]. Values of the hyper-parameter are presented in Table 3.

## 5. Evaluation

The procedure of the experiment used to identify learning styles has been described in Section 1.1.5. All experimental details have been described in our past study [27]. In this paper, we used EEG data to verify our TSMG method.

### 5.1. Analysis of Effectiveness of Multi-Scale Convolution

**Figure 8:**
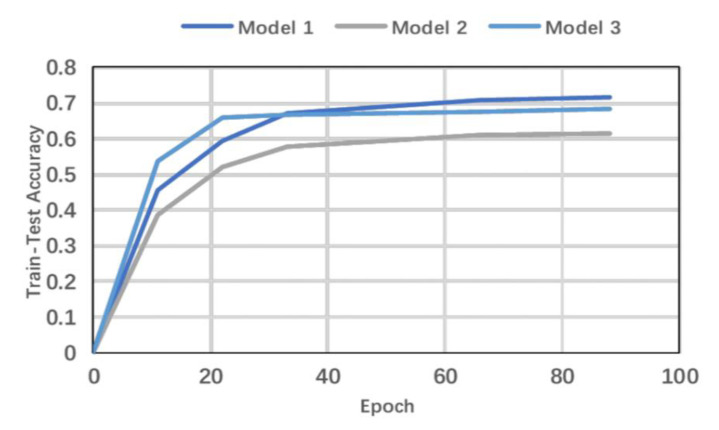
Variations in the training accuracy of three models.

To verify the effectiveness of adding multi-scale features as well as the impact of model depth on the accuracy of recognition of EEG signals, three model structures were designed for comparison in the experiment. They were as follows: (1) Model 1 was the TSMG model proposed in this paper. (2) Model 2 was a common convolutional neural network (CNN) model that did not have multi-scale feature extraction and used only 3 × 3 convolution kernels. (3) Model 3 was a multi-scale CNN model with five modules. Figure 8 shows changes in the curve of accuracy of the three model structures during the training process.

A comparison among the models shows that Model 2, which did not have multi-scale features during training, always had a lower accuracy than Models 1 and 3. Although Model 3 converged more quickly than Model 1, with an accuracy of 68.3% after 50 epochs of training, its overall accuracy was not as good as that of Model 1.

Thus, Model 1, the proposed model, achieves the highest accuracy, 72.35%, at recognizing learning styles when it is trained for 60 epochs. The results show that adding multi-scale features can improve the ability of the convolution layer to express EEG data and improve the accuracy of recognition. Although increasing the depth of the model can reduce the number of epochs required for training, overfitting occurred and reduced the accuracy of classification if the model was too deep. This experiment verifies that the TSMG model had the best recognition effect at a higher training speed than the other models. Compared with the model without multi-scale features, its accuracy was higher by 9.8%.

### 5.2. Analyzing Effectiveness of 1D Convolution

To verify improvements in the speed of calculation and the quality of the parameters made when using the TSMG model, we designed the following method of comparison: using the same model structure, a 2D convolution was used to replace the 1D convolution and construct a 2D CNN model for comparison with the proposed model.

The rule for calculating the training parameters of the convolution model is as follows:

(7) $$P_{i}=h_{i}w_{i}c_{i-1}k_{i}$$

where $P_{i}$ represents the training parameters of the convolutional layer *i*, $h_{i}$ is the length of the convolution kernel, $w_{i}$ is its width, $c_{i-1}$ is the number of convolution kernels in the previous layer, and $k_{i}$ is the number of convolution kernels.

In terms of the number of training parameters, by using the 1D convolution kernel, the number of parameters required for training was reduced from 99,810 to 57,960 compared with the 2D CNN, and the amount of requisite calculation was significantly reduced by 41.93%.

### 5.3. Analysis of Overall Accuracy

Given the high inter-subject variability of the EEG signal, leave-one-out cross-validation [68] is implemented, that is, one subject is selected at a time as the test set, and the remaining 13 subjects are used as the training set, so that the training samples and test samples are from different subjects.

We performed leave-one-out cross-validations on the TSMG, SVM, KNN, BP, VGGNet, and ResNet to compare them in terms of average accuracy of recognition. The results are shown in Table 4. The accuracy of the TSMG on the test set was 72.65%, higher than those of the SVM (63.18%), BP (59.32%), KNN (52.73%), VGGNet (65.28%), and ResNet (68.31%).

### 5.4. Visualizing Intermediate Results

**Figure 9:**
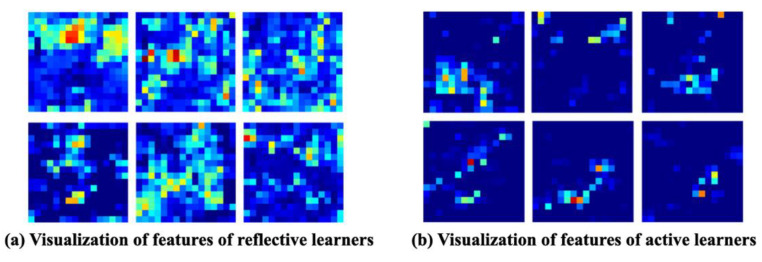
Visualization of features of the learners’ learning styles.

Visualizing the output of features of the model can help enhance the interpretability of the results of recognition, thereby making possible a more intuitive analysis of network representations, diagnoses of the results of training, and improvements in network design [69]. Moreover, feature visualization can be used for feature analysis and the interpretation of the results once the model has been trained, without modifying or retraining it. The feature visualizations are presented in the form of a heat map. A heat map is an image composed of colors of different intensities, where the intensity of a given color of a pixel corresponds to its importance. From a mathematical point of view, a heat map is a set of importance values corresponding to input variables. Each element in the set represents the correlation between its corresponding input variables and the output. We used the EEG training data of six reflective learners and six active learners to render a feature map of EEG signals in the last layer of model training. Figure 9 shows that there were prominent differences between EEG data of the learning styles after feature extraction through model training. Therefore, it is verified that TSMG model achieves good results on feature extraction of EEG data with learning style features.

### 5.5. Analyzing Contribution of EEG Leads

To verify the correlation between different EEG leads and learning styles, and to compare the accuracy obtained with different positions of the electrode, we screened out the response data for a typical active learner (Act-Set), a typical reflective learner (Ref-Set), and the overall dataset (Avg-Set) of 14 subjects to form three training datasets. The relevant contributions to the leads were then analyzed.

For the above three training datasets (Act-Set, Ref-Set, and Avg-Set), we first remove the data of 1 channel in turn from the data of all 14 channels, so as to obtain the lacking channel set except for the removed channel (for example: remove F3 channel from the 14 complete acquisition channels, only use the remaining 13 channels for recognition, and name them as EXC-F3).

The three datasets (Act-Set, Ref-Set, and Avg-Set) were trained separately, and each used the 14 13-channel sets, with one channel removed, as input features.

**Figure 10:**
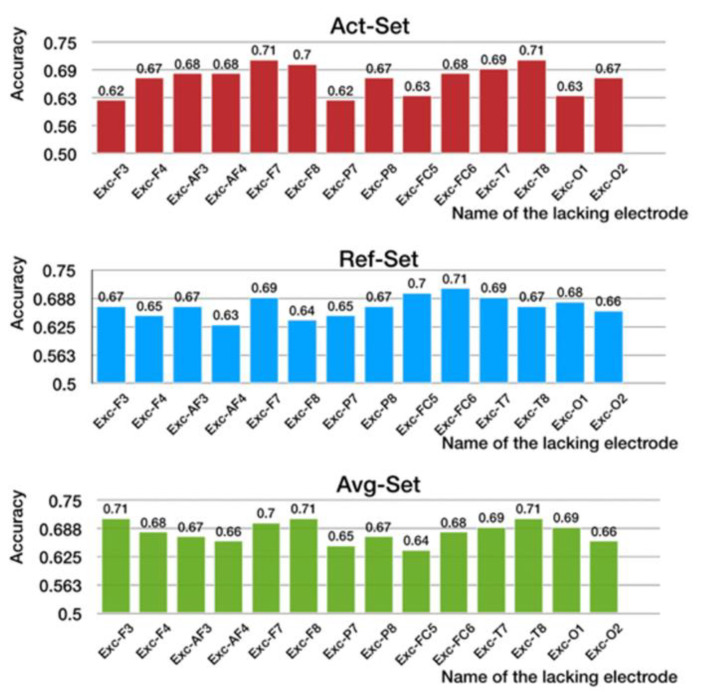
Accuracy of recognition on three datasets (Act-Set, Ref-Set, Avg-Set).

The results are shown in Figure 10. For Act-Set, it is clear that the accuracy of the model decreased most significantly following the removal of channel F3 or P7. Therefore, F3 and P7 were highly correlated with active learning style.

Therefore, the results show that the EEG leads that were highly correlated with active learning were P7, FC5, O1, and F3. The leads correlated with reflective learning were AF4, F8, P7, and F4, and those highly correlated with the students’ styles were FC5, P7, AF4, and O2. Therefore, we can select EEG leads for different positions in experiments according to different scenarios of learning styles in future work.

### 5.6. Statistical Hypothesis Test of Accuracy

We compared the recognition accuracy of the TSMG model and the other models by using the Wilcoxon signed-rank test [70]. To answer the question of whether the accuracy of recognition of the TSMG model was significantly higher than those of the other models (i.e., SVM, BP, KNN, VGGNet, and ResNet), we propose the hypotheses shown in Equations (8) and (9), where P(TSMG) and P(i) are the median values of the accuracy of recognition of the TSMG model and the other models, respectively:

(8) H0: P(TSMG)> P(i),   i= SVM, BP, KNN, VGGNet, ResNet

(9) H1: P(TSMG)≤ P(i),   i= SVM, BP, KNN, VGGNet, ResNet

The results of the Wilcoxon test are shown in Table 5. Hypothesis H0 in Equation (8) was rejected, and H1 in Equation (9) was accepted. This indicates that the accuracy of recognition of the TSMG model was significantly higher than the other models. There were statistically significant differences in the accuracy of recognition of subjects with different learning styles. This further verifies the design of the proposed model.

## 6. Conclusions

In this paper, we proposed a model called the TSMG to identify people’s learning styles efficiently and accurately by using EEG signals. It includes a non-overlapping sliding window, a 1D spatio-temporal convolution, multi-scale feature extraction, global average pooling, and group voting mechanism.

The non-overlapping sliding window can process information of any length to extract the characteristics of each item of data, and can minimize the amount of requisite calculation to improve efficiency. The temporal and spatial convolutions can extract the temporal and spatial characteristics of EEG signals. They can also reduce the cost of calculation of the model. The 1D convolution can significantly reduce the number of parameters needed, and the multi-scale parallel convolution structure can enhance the feature abstraction capability of the model. The global average pooling strategy was used to reduce the number of training parameters of the model and overcome the limitation of the traditional fully connected layer regarding the size of input data. Based on the idea that collective decision-making ability is superior to individual decision-making ability, the group voting mechanism was used to improve the accuracy of recognition of the model.

We visualized the intermediate results of the proposed model to intuitively grasp its efficiency of recognition of learning styles, and analyzed the effectiveness of EEG leads. The proposed TSMG model also has significance for the recognition of EEG data in other fields.

We also created a dataset of EEG data containing the features of learning styles (called the LSEEG dataset). It can be used in relevant research to better evaluate models used to identify EEG signals.

## Figures and Tables

**Table 1:** Details of multi-scale feature extraction module.

Model Configuration					
A		B		C	D
Input					
1 × 1 Conv. (10)		1 × 1 Conv. (10)		1 × 3 Conv. (20)	1 × 1 Conv. (40)
Batch Normalization					
ReLU					
1 × 7 Conv. (20)		1 × 5 Conv. (20)		3 × 1 Conv. (40)	
Batch Normalization					
ReLU					
7 × 1 Conv. (40)	3 × 1 Conv. (40)	5 × 1 Conv. (40)	3 × 1 Conv. (40)		
Batch Normalization					
ReLU					
Concatenation					

**Table 2:** Feature extraction methods for comparison models.

EEG Feature Type	Feature Index	Notation of the Extracted Feature
Frequency-domain features	No. 1–56, power-related features	Mean power for all EEG channels (F3, F4, AF3, AF4, F7, F8, P7, P8, FC5, FC6, T7, T8, O1, and O2) in the theta (4–8 Hz), alpha (8–12 Hz), beta (12–30 Hz), and gamma (30–45 Hz) bands (14 channels × 4 power features = 56 features).
	No. 57–76, power-difference-related features	Differences in mean power in 14 EEG channel pairs between the right and left scalps (F4-F3, AF4-AF3, T8-T7, P8-P7, and O2-O1) in the theta, alpha, beta, and gamma bands (5 channel pairs × 4 power differences = 20 features).
Time-domain features	No. 77–174, time-domain-related features	Mean, variance, zero crossing rate, Shannon entropy, spectral entropy, kurtosis, and skewness of 14 EEG channels (7 features × 14 channels = 98 features).

**Table 3:** Parameter settings of the SVM, BP, KNN, VGGNet, and ResNet.

Classification Method	Hyper-Parameter Settings
SVM	Regularization and number of kernel parameters: 16, 128.
BP	Numbers of hidden nodes, layers and training epochs: 144, 4, 60.
KNN	k value: 26
VGGNet	Number of convolution layers, batch size, optimizer, and number of training epochs: 3, 30, Adam, 100
ResNet	Number of convolution layers, fully connected layers, basic block size, batch size, optimizer, learning rate, number of training epochs: 17, 1, 3 × 3, 30, SGD, 0.1, 100

**Table 4:** Leave-one-out cross-validation on the TSMG, SVM, KNN, BP, VGGNet, and ResNet.

Classifier	Leave-One-Out Cross-Validation Accuracy (%)														
	F-1	F-2	F-3	F-4	F-5	F-6	F-7	F-8	F-9	F-10	F-11	F-12	F-13	F-14	LOO Average (%)
TSMG	78.56	76.21	72.35	75.68	73.24	71.62	72.69	70.81	69.72	73.54	67.28	68.34	73.61	73.50	72.65
SVM	64.23	65.37	61.20	65.37	63.28	64.78	60.43	61.57	62.78	63.12	66.31	60.39	61.24	64.57	63.18
BP (3 h-layers)	61.56	60.37	59.24	58.30	57.56	60.58	58.36	57.98	58.36	61.58	61.25	57.58	59.42	58.38	59.32
BP (5 h-layers)	58.64	60.31	57.12	56.36	58.64	56.84	56.36	61.47	58.71	57.62	56.55	58.12	57.23	56.89	57.91
KNN	53.64	52.57	55.61	50.47	51.84	50.67	54.87	51.37	55.67	54.32	51.64	50.47	52.78	52.31	52.73
VGGNet	68.54	65.71	66.80	67.52	67.91	64.33	62.41	64.56	64.81	62.72	66.23	65.34	62.65	64.40	65.28
ResNet	71.31	68.43	65.67	70.54	68.40	72.62	67.32	66.31	67.42	65.14	68.47	67.24	70.21	67.30	68.31

**Table 5:** Results of Wilcoxon signed rank test.

i	W Statistics	*p*-Value
SVM	105.0	6.10352 × 10^−5^
BP (three-layer)	105.0	6.10352 × 10^−5^
BP (five-layer)	105.0	6.10352 × 10^−5^
KNN	105.0	6.10352 × 10^−5^
VGGNet	105.0	6.10352 × 10^−5^
ResNet	101.0	0.00043

## Data Availability

The original contributions presented in the study are available in https://github.com/aegine-lab/dataset, accessed on 23 October 2021.
